# Stable introduction of *Wolbachia w*Pip into invasive *Anopheles stephensi* for potential malaria control

**DOI:** 10.1371/journal.pntd.0012523

**Published:** 2024-09-26

**Authors:** Yongkang Liang, Julian Liu, YiLian Wu, Yu Wu, Zhiyong Xi

**Affiliations:** 1 Department of Parasitology, Key Laboratory of Tropical Disease Control of the Ministry of Education, Sun Yat-sen University, Guangzhou, China; 2 Guangzhou Wolbaki Biotech Co., Ltd, Guangzhou, China; 3 Department of Microbiology, Genetics, & Immunology, Michigan State University, East Lansing, Michigan, United States of America; QIMR Berghofer Medical Research Institute, AUSTRALIA

## Abstract

The spread and invasion of the urban malaria vector *Anopheles stephensi* has emerged as a significant threat to ongoing malaria control and elimination efforts, particularly in Africa. The successful use of the maternally inherited endosymbiotic bacterium *Wolbachia* for arbovirus control has inspired the exploration of similar strategies for managing malaria vectors, necessitating the establishment of a stable *Wolbachia-Anopheles* symbiosis. In this study, we successfully transferred *Wolbachia w*Pip into *An*. *stephensi*, resulting in the establishment of a stable transinfected HP1 line with 100% maternal transmission efficiency. We demonstrate that *w*Pip in the HP1 line induces nearly complete unidirectional cytoplasmic incompatibility (CI) and maintains high densities in both somatic and germline tissues. Despite a modest reduction in lifespan and female reproductive capacity, our results suggest the *Wolbachia* infection in the HP1 line has little impact on life history traits, body size, and male mating competitiveness, as well as the ability of its larvae to tolerate rearing temperatures up to 38°C, although *w*Pip densities moderately decrease when larvae are exposed to a constant 33°C and diurnal cyclic temperatures of 27–36°C and 27–38°C. These findings highlight the potential of the HP1 line as a robust candidate for further development in malaria control.

## Author summary

Recent efforts have successfully utilized the endosymbiotic bacterium *Wolbachia* to control mosquito-transmitted viral diseases like dengue in multiple countries. However, similar initiatives have been limited in combating malaria, the most devastating and deadly mosquito-borne disease, which claims over half a million lives each year. This is primarily due to the difficulty in establishing a stable, maternally inheritable *Wolbachia* infection in *Anopheles*, the mosquito vector responsible for malaria transmission. A significant concern in malaria control is the invasion of the urban malaria vector *Anopheles stephensi* into Africa, where malaria burden is highest, and over 40% of the population resides in urban areas. Building on the previous breakthrough of establishing *Wolbachia* strain *w*AlbB in *An*. *stephensi*, the author has now achieved a second stable infection by transferring the *w*Pip strain from *Culex pipiens* into this mosquito species using embryonic microinjection. The resultant transinfected HP1 line induces nearly complete cytoplasmic incompatibility when crossed with wild mosquitoes, displays robust fitness and male mating competitiveness, and exhibits strong resilience against heat stress. These advantageous traits position the HP1 line as a promising candidate for further development in malaria control.

## Introduction

Malaria, caused by *Plasmodium* spp., which are transmitted by *Anopheles* mosquito species [1], imposes a significant public health burden [2]. The World Health Organization reported approximately 249 million malaria cases and 608,000 related deaths across 85 endemic countries in 2022, a substantial increase from pre-COVID-19 pandemic estimates [3]. Despite the availability of artemisinin-based treatments, the emergence and spread of resistant *Plasmodium* strains and the absence of an effective vaccine have significantly hindered the global malaria eradication efforts [4–6]. *Anopheles stephensi*, capable of thriving in urban and man-made environments and transmitting both *P*. *falciparum* and *P*. *vivax* [7,8], is native to South Asia and parts of the Arabian Peninsula but has recently expanded its range into sub-Saharan Africa. This region, where malaria’s burden is most severe and over 40% of the population resides in urban areas, has become a critical focus for mosquito control efforts. A recent WHO initiative underscores the urgency of developing innovative control strategies targeting *An*. *stephensi* [9]. Currently, control efforts primarily rely on chemical pesticides; however, the rapid development of resistance among vectors and the adverse environmental impacts of these insecticides [10,11], necessitate the development of new methods for vector control.

The maternally transmitted endosymbiotic bacterium *Wolbachia pipientis* naturally infects ~40% of all terrestrial insect species [12]. Native *Wolbachia* infections have been discovered in several anopheline populations across Africa [13–17]. Unfortunately, except for one report of high-density infection [16,18], other native *Wolbachia* infections have been reported with low prevalence and low titer, which has led to controversy, as these could reasonably result from contamination or other environmental sources. Also, none of these strains has been observed to induce cytoplasmic incompatibility (CI), the early embryonic death that occurs when infected males mate with females that are either uninfected or infected with a different *Wolbachia* strain [19]. Stable, artificial *Wolbachia* infections can be generated in mosquitoes through embryo microinjection. Although many *Wolbachia*-transinfected *Aedes* lines have been developed through this technique, thus far only a single *Wolbachia* transinfected anopheline line has been generated: the *An*. *stephensi* LB1 line carrying *w*AlbB strain originally from *Aedes albopictus* [20]. Stable transinfections typically cause CI in mosquitoes, which acts as a type of natural genetic drive to allow *Wolbachia* to spread rapidly into uninfected mosquito populations [21]. *Wolbachia* infection can also cause pathogen blocking, which makes mosquitoes resistant to infection with key pathogens and decreases the likelihood that these pathogens will be transmitted [22–24]. In LB1 line, *w*AlbB was found to induce *An*. *stephensi* refractory to both the human malaria parasite *Plasmodium falciparum* and the rodent parasite *P*. *berghei* [20,25].

Strategies that utilize *Wolbachia* to control arbovirus transmission have shown considerable advancement, with successful field trials demonstrating population reductions [26,27] and reduced disease transmission through the replacement of pathogen-susceptible populations with *Wolbachia*-infected pathogen-resistant ones [21,28–30]. Similar efforts are underway in Hawaii, targeting *Culex* mosquitoes to control avian malaria [31]. However, the application of *Wolbachia* for malaria control is less developed, primarily because only one *Wolbachia*-transinfected *Anopheles* line was available until recently, and major resources were focused on developing *Wolbachia* for dengue control. Consequently, there is a need to develop additional transinfected lines, identify those with substantial parasite-blocking potential and minimal fitness costs, and assess the feasibility of a *Wolbachia*-based population suppression strategy to eradicate this invasive species and restore the original *An*. *stephensi*-free ecosystems in urban Africa.

In this study, we established the *An*. *stephensi* HP1 line transinfected with *w*Pip, originally derived from *Culex pipiens molestus*. In the HP1 line, *w*Pip exhibits perfect maternal transmission and induces nearly complete CI in crosses between infected males and uninfected wild females. While *w*Pip in the HP1 line has little impact on mosquito fitness and shows tolerance to the rearing temperatures up to 38°C, it experiences a modest reduction in lifespan and female reproductive capacity, with *w*Pip densities moderately decreasing when larvae are exposed to a constant 33°C and diurnal cyclic temperatures of 27–36°C and 27–38°C. These findings underscore the robustness of the HP1 line and its potential utility in vector control strategies.

## Methods and materials

### Ethics statement

This study was conducted in accordance with the recommendations of the Animal Care and Use Committee (ACUC) of Sun Yat-sen University. Mice were used for mosquito rearing according to protocol SYSU-IACUC-2022-B0023, approved by the Institutional Animal Care and Use Committee (IACUC) of Sun Yat-sen University.

### Mosquito colonies

Originally collected from India, the *An*. *stephensi* Hor line was provided by Professor Wen-Yue Xu [32]. The *Ae*. *albopictus* transinfected GTM line was established by transferring *Wolbachia w*Pip from *Cx*. *pipiens molestus*, a gift from Professor Tongyan Zhao, into a tetracycline-treated *Ae*. *albopictus* Guangzhou line through embryonic microinjection in our laboratory in 2014. Since then, *w*Pip has stably maintained 100% maternal transmission in the GTM line. Both *An*. *stephensi* line and GTM line were maintained on 10% sugar solution at 27 ± 1°C, 70 ± 10% with a 12-hr L/D (light/dark) cycle [20]. Five to seven days old female mosquitoes were fed on the blood of anesthetized mice to initiate egg development.

### Embryonic microinjection

Embryonic microinjection involved transferring cytoplasm from GTM embryos into the posterior of recipient Hor embryos, following previously reported procedures with minor modifications [20,33]. Post-injection, embryos were incubated at 70% relative humidity and 27°C for approximately 40 minutes, then removed from oil (Halocarbon oil 700, Simga) and placed on wet filter paper supported by water-soaked cotton. Hatched larvae were reared under standard maintenance conditions as described earlier. The establishment of an isofemale line and screening for stable transinfection followed methods previously detailed [20,33]. Briefly, G0 females were isolated as virgins and mated with Hor males. After oviposition, G0 parents were tested for *Wolbachia* infection using the universal primers 81F and 691R [34], based on established protocols [20,26]. G0 females testing negative for *Wolbachia* were discarded along with their progeny. This screening process was repeated in each generation until all offspring tested positive for *Wolbachia* infection. After nine generations of screening, we established the transinfected *An*. *stephensi* HP1 line with a stable *Wolbachia* infection, which has been outcrossed with Hor for six generations to homogenize the host genetic background before further study.

### CI crosses

At G30, standard crosses were conducted to evaluate the patterns and intensity of cytoplasmic incompatibility (CI) [20]. Each cross involved three replicates, consisting of 10 virgin females and 10 virgin males. Adults had constant access to a 10% sucrose solution. Mating was allowed for five days before the females were blood-fed on anesthetized mice. Two days post-blood meal, oviposition cups lined with wet filter paper were placed in the cages. Females were given 2 to 3 days to lay eggs on the filter paper. Egg hatch rates were assessed two days post-hatching under a dissecting microscope.

### PCR assay of *Wolbachia* densities in the whole bodies and different tissues of HP1 line

qPCR was conducted to assess *Wolbachia* densities in the whole bodies, ovaries, midguts, fat bodies, salivary glands of 7-day-old females and the whole bodies of 7-day-old males, with five replicates for each tissue or whole body. All the tissues were dissected in 1× PBS solution, and DNA from these tissues was extracted individually following previously described methods [20]. *Wolbachia* density for each sample was quantified using ChamQ SYBR qPCR Master Mix (Without ROX). The assay targeted the *Wolbachia wsp* gene and the host ribosomal protein S6 (*rps6*) gene using specific primers. The primers for *rps6* gene were as previous described [20], and those for *w*Pip *wsp* gene were: *w*Pip F: TATTTCCCACTATATCCCTTC; *w*Pip R: GGATTTGACCTTTCCGGC [26]. Two recombinant plasmids containing the targeted gene fragments were serially diluted to construct separate standard curves for the *wsp* and *rps6* genes.

### Immature development

For both HP1 line and Hor line, larval development and survival were determined as previously described [35]. Firstly, 150 larvae (< 2h old) were transferred to a plastic container (20 * 10 * 5 cm) filling with 1,000 ml distilled water. Bovine liver powder solution at a concentration of 60 g/L was provided daily. Three biological replicates were performed for each group. Pupae were collected daily at 9:00 am and 4:00 pm and transferred to culture tubes. Adult emergence was also recorded daily at 9:00 am, 12:00 am and 3:00 pm. Time to pupation and time to emergence were both recorded as the time required for the development of L1 to pupa and L1 to adult stages, respectively. Survival to pupation and survival to adult emergence were calculated as the proportion of larvae that survived from L1 to pupal stage and from L1 to adult stage, respectively.

### Wing length

To assess adult size, the left wing of 29 males and 29 females from each line was collected for measurement under a microscope (Olympus CX31, Japan). The wing length was determined by measuring the distance from the distal edge of the alula to the end of the radius vein (excluding fringe scales) [35]. Three replicates of 29 males and 29 females each were conducted per line.

### Adult longevity

Adult longevity was assessed as previously described with slight modifications [35]. Twenty-five newly emerged males and females each were placed in 15 × 15 × 15 cm stainless steel cages, with continuous access to a 10% sugar solution. Adult longevity was assessed under four different feeding regimes: (a) virgin males receiving only sugar solution, (b) virgin females receiving only sugar solution, (c) mated females receiving only sugar solution, and (d) mated females receiving both sugar solution and weekly blood meals that were repeated more than four times. Three biological replicates were conducted for each feeding regime. Mosquito mortality was monitored daily; dead individuals were counted and removed to determine longevity, continuing until all mosquitoes in each cage had perished.

### Female fecundity and fertility

To assess female fecundity and fertility, 6- to 7-day-old females from the maintenance colonies were randomly selected and placed into a new 23 × 23 × 23 cm stainless steel cage [20,35]. After providing a blood meal, unfed mosquitoes were removed. Two days later, thirty-one individual females were transferred to 50-ml tubes lined with wet filter paper at the bottom for egg collection. After collecting eggs for two nights, the females were removed. The oviposition filter papers were transferred the next day to a hatching cup with fresh water and some larval food, and allowed to hatch over two days. Then the hatched eggs and the total number of eggs were scored under a microscope.

### Male mating competitiveness assay

The mating competitiveness of HP1 and Hor males with Hor females was assessed with minor modifications from previous methods [35]. Before the experiment, males and females were placed separately in laboratory cages (23 × 23 × 23 cm). For the assay, males were released into large cages one hour before introducing the females. Twenty 1–2 day-old Hor females were paired with 2–3 day-old Hor and HP1 males in varying ratios: 1:0, 1:1, 1:3, 1:5, and 0:1, with three to five biological replicates conducted for each ratio. Mating was allowed for five days before the females were blood-fed on anesthetized mice. Two days post-blood meal, oviposition cups lined with wet filter paper were placed in the cages. The females were given 2 to 3 days to lay eggs on the filter paper. Egg hatch rates were assessed two days post-hatching under a microscope as described above, and compared against an expected rate based on the assumption of equal mating competitiveness between HP1 and Hor males under random mating conditions. The Fried male mating competitiveness index was calculated following established methods [36].

## Temperature sensitivity studies under laboratory conditions

Two temperature regimens were used to evaluate the tolerance of HP1 line to high temperatures in term of their impacts on *Wolbachia* density and mosquito immature development. In the first regimen, first-instar larvae of HP1 line and Hor line were exposed to temperature maintained constantly at 30°C, 33°C, 36°C and 38°C up to pupal stage. In the second regimen, the larvae were reared at diurnal cyclic temperatures of 27–33°C, 27–33°C, 27–36°C and 27–38°C to pupae. Batches of 30 first-instar larvae of each strain were released separately into a plastic container (20 * 10 * 5 cm) filling with 300 mL distilled water and the containers were placed inside an artificial climate Chamber till the larvae pupated. The water baths were set to maintain temperature constantly at 30°C, 33°C, 36°C and 38°C or at daytime cycling temperatures of 27–33°C, 27–33°C, 27–36°C and 27–38°C. Three replicates (each with 30 larvae) were kept for each temperature regimen and for each strain. Simultaneously, larvae of HP1 line and Hor line were maintained constantly at 27 ± 1°C as controls for each experiment. The larvae were fed with Bovine liver powder solution as described above. The water temperature was recorded using submerging data loggers. Pupae were collected daily at 9:00 am and 3:00 pm and transferred to 15 ml culture tubes containing 1 ml of water. All tubes were kept at a temperature of 27°C. Adult emergence was also recorded daily at 9:00 am and 3:00 pm. Survival to pupation and survival to adult emergence were calculated as the proportion of larvae that survived from L1 to pupal stage and from L1 to adult stage, respectively. Five-day-old adults emerged from all the treatment were used to detect the *Wolbachia* frequency and density by qPCR as described above [20,26].

### Statistical analysis

All data were analyzed using Graphpad Prism 9.0 or SPSS 26 software. Kolmogorov-Smirnov tests were used to analyze whether the data obeyed normal distribution. Student’s *t* test or Mann-Whitney *U* test was then used to analyze the significant difference in fecundity and fertility of female mosquitoes, *Wolbachia* density of females and males, survival assays and wing length. The life span of adult mosquitoes was analyzed using Log-rank (Mantel-Cox) tests, and the comparison between the experimental and predicted mating competitiveness of males was analyzed using one sample *t* test. ANOVA, followed by Tukey post-hoc tests, was used to analyze the significant difference in CI cross, *Wolbachia* density in female tissues, and temperature sensitivity of the HP1 line.

## Results

### Generation of the HP1 line

**Fig 1:**
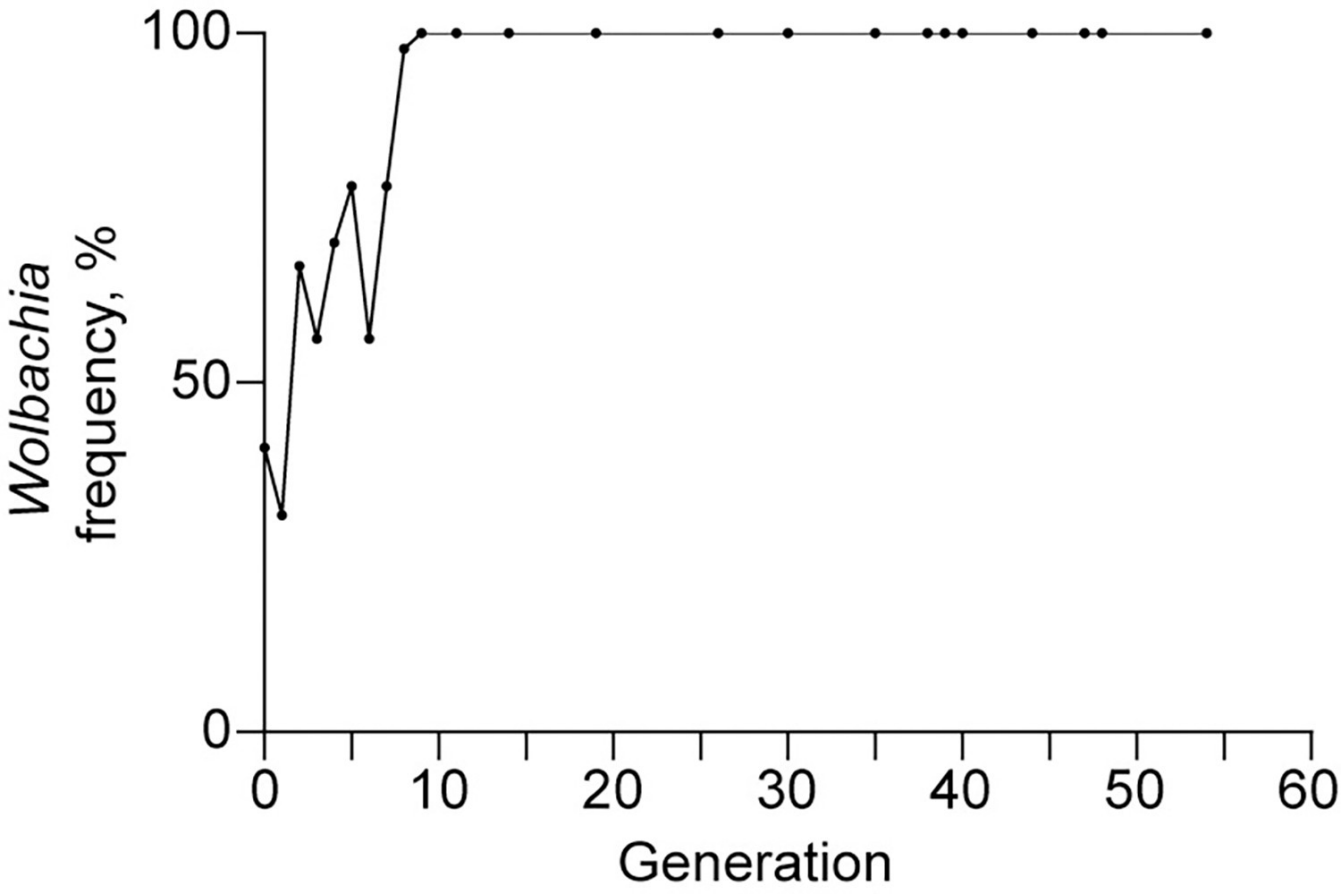
Generation of a stable *w*Pip transinfection in *An*. *stephensi* through intensive PCR-screenings over multiple generations. In each generation, 8 to 16 female parents from each isofemale line were individually assayed by PCR to detect *w*Pip infection after producing offspring. Offsprings from positive mothers were pooled to establish subsequent generations for further screening. Generation 0 consists of individuals that survived embryonic microinjection.

In order to establish novel *Wolbachia* transinfection in *An*. *stephensi*, we transferred *Wolbachia* from *w*Pip-transinfected *Ae*. *albopictus* GTM line into *An*. *stephensi* wild-type Hor line by embryonic microinjection. There were 81 larvae hatched from 920 Hor embryos injected, resulting in 68 pupae and 59 adults (G0) survived (Table 1). Screening of those adults detected *Wolbachia* infections in 44.1% and 36.0% of males and females, respectively. Individual isofemale line was established from each of 9 infected G0 females by outcrosses with Hor males and only three of them produced G1 offsrpings. We observed only one isofemale line successfully inherited *w*Pip into G1 progenies, achieving approximately 30% maternal transmission efficiency (Tables 1 and S1). Three positive G1 females were outcrossed with Hor males to establish the next generation and *Wolbachia* infections were detected in 71.4% and 60.0% of male and female G2 progenies, respectively (Table 1). The positive female offspring were individually crossed with Hor males to obtain their next generations and screen was repeated until 100% maternal transmission efficiency was observed at both G9 and G10 (S2 Table). This transinfected line, which was outcrossed with Hor for six generations (from G1 to G6), is hereafter referred to as the HP1 line. The stability of maternally inherited *w*Pip infection in HP1 was confirmed by PCR assay of randomly selected individuals at the subsequent generations until G55 (the last generation assayed in this study thus far) (Figs 1 and S1 and S2 Tables). Thus, we succeeded in establishing the second *Wolbachia* transinfection in *Anopheles*, following the previous LB1 line transinfected with *w*AlbB.

**Table 1:** Survival of microinjected *An*. *stephensi* embryos and the resulting *Wolbachia w*Pip infection status in the G0, G1 and G2 adults.

Hatch^#^ rate, %	Pupation^#^ rate, %	Eclosion^#^ rate, %	%, *w*Pip infection frequency					
			G0 ♂	G0 ♀	G1 ♂	G1 ♀	G2 ♂	G2 ♀
8.8 (81/920)	84.0 (68/81)	84.0 (59/68)	44.1 (15/34)	36.0 (9/25)	31.6 (6/19)	30.0 (3/10)	71.4 (5/7)	60.0 (3/5)

^**#**^Note: Hatch rate (%) = Larvae/injected eggs, Pupation rate (%) = Pupae/larvae, Eclosion rate (%) = Adult/pupae.

### CI crosses and *Wolbachia* densities across whole bodies and different tissues of HP1 mosquitoes

**Fig 2:**
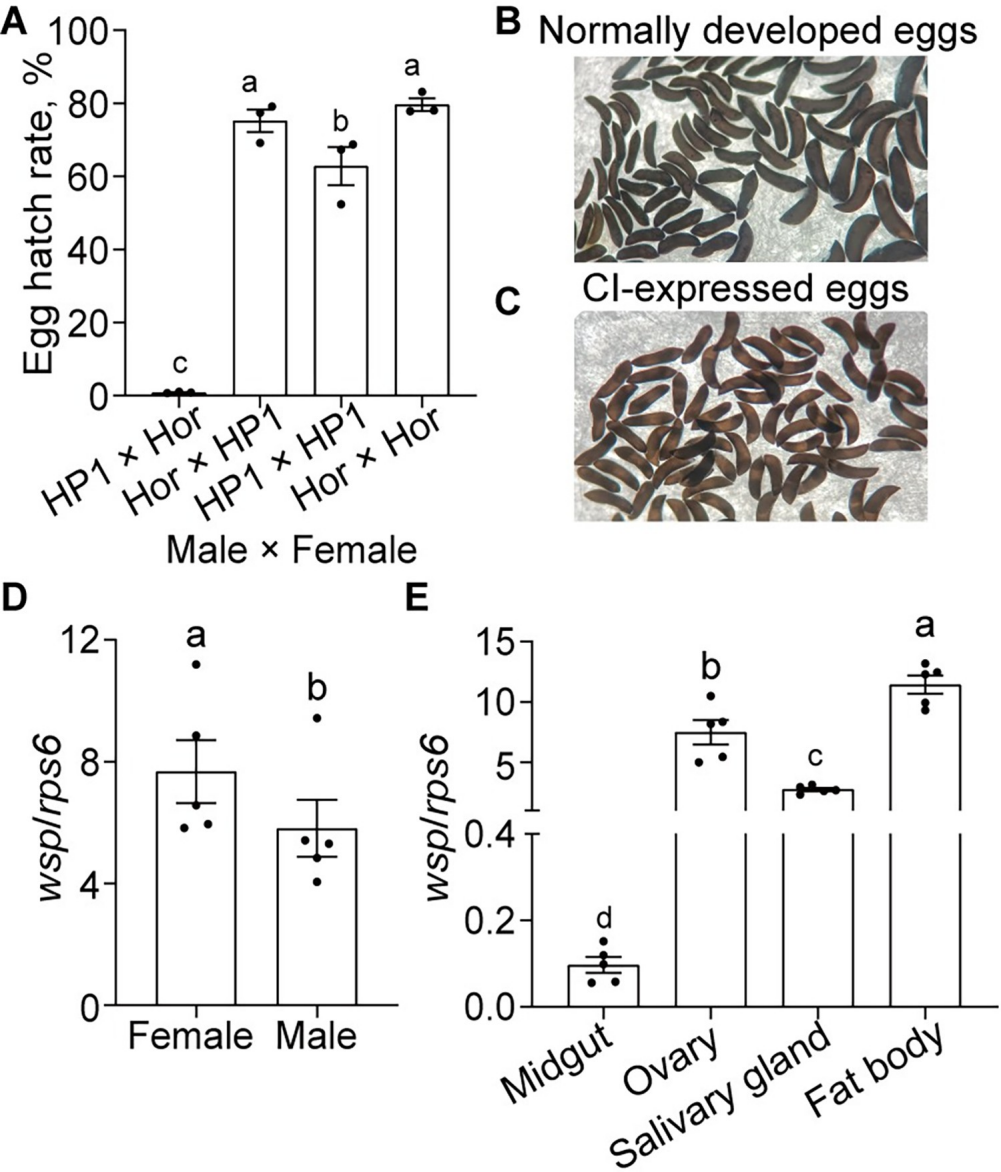
CI crosses and *Wolbachia* densities in the whole bodies and different tissues of HP1 mosquitoes. (A) Results of reciprocal crosses between the HP1 line and wild non-infected Hor line. (B) *An*. *stephensi* embryos in the compatible crosses with normal development. (C) *An*. *stephensi* embryos in the CI crosses with the early death during development. (D) Relative densities of *w*Pip in HP1 females or males at 7 days old. (E) Relative density of *w*Pip in ovaries, midguts, fat bodies, salivary glands of 7-day-old non-blood-fed HP1 females. *wsp* and *rps6* genes were used as target and host reference genes, respectively. Error bars represent the standard errors based on 3 biological replicates for A and 5 biological replicates for D and E. Different lowercase letters above each column indicate significant differences, *P* < 0.05, Student’s *t* test for D, ANOVA analysis, followed by Tukey post-hoc tests for A and E.

To determine whether *w*Pip induces CI in *An*. *stephensi*, we set up the crosses between transinfected HP1 and wild-type Hor lines. Out of 1,992 eggs resulting from crosses between HP1 males and Hor females, nearly no egg hatched (0.8 ± 0.1%), whereas HP1 females rescued CI when mating with HP1 males, with the egg hatch rates (62.9 ± 5.2%), slightly lower than both the self-crosses of Hor (79.6 ± 1.7%) and compatible crosses between HP1 females and Hor males (75.5 ± 3.1%) (Fig 2A and S3 Table, ANOVA analysis, F_3, 8_ = 133.3, *P* < 0.05). These results are similar to the previous observation in *w*AlbB-infected LB1 line, indicating a typical pattern of unidirectional CI induced by *w*Pip in *An*. *stephensi*. In contrast to the normal embryonic development with dark black pigment, CI embryos stayed at light color, indicating deficiency in eggshell melanization (Fig 2B and 2C).

To examine the densities of *w*Pip across different tissues in HP1, we collected the whole bodies of both sexes as well as the midguts, salivary glands, ovaries and fat bodies of 7-day-old non-blood-feeding females, and measured the numbers of *w*Pip genome copies using real-time qPCR. The results showed that *w*Pip densities in the whole bodies of females were significantly higher than in the males (Fig 2D, Student’s *t* test, *P* < 0.05). Furthermore, the fat bodies exhibited the highest *w*Pip densities among all the tissues, followed by ovaries and salivary glands, with the lowest *w*Pip densities in midguts (Fig 2E, ANOVA analysis, F_3, 16_ = 62.34, *P* < 0.05). These results indicate that *w*Pip has distinct tissue tropism, being highly enriched in fat bodies but only minimally present in midguts.

### Impact of *w*Pip on the development and body size of HP1 line

To examine the impact of *w*Pip on the development and body size of *An*. *stephensi*, we assessed life history traits and adult body sizes of HP1 and Hor lines. There were no significant differences in survivorship and development time from the L1 larval stage to pupae/adults, sex ratios, or wing lengths of both females and males between the HP1 and Hor lines (Table 2). These results indicate that, similar to observations in the LB1 line, there are no measurable fitness costs associated with immature development and adult body size in *An*. *stephensi* due to *w*Pip.

**Table 2:** Life history traits and adult body sizes of HP1 and Hor lines.

Parameters	HP1	Hor	Z value	Df	*P*-value
Survivorship from larvae to pupae, %	96.44 ± 1.2	96.00 ± 1.7	-0.2181	4	0.8380
Pupation time, hr	164.67 ± 0.3	165.33 ± 0.9	0.7071	4	0.5185
Survivorship from larvae to adult, %	92.89 ± 1.4	94.44 ± 2.0	-0.6498	4	0.5513
Sex ratio^a^, %	58.85 ± 0.3	47.78 ± 2.5	-4.364	4	0.1000
Male emergence time, hr	176.67 ± 1.2	182.00 ± 6.0	0.8677	4	0.4345
Female emergence time, hr	190.67 ± 4.4	193.67 ± 4.5	0.4790	4	0.6569
Male wing length, mm	2.874 ± 0.0052	2.877 ± 0.0048	0.4442	172	0.6575
Female wing length, mm	3.113 ± 0.0054	3.125 ± 0.0045	1.762	172	0.0798

^a^ Sex ratio was calculated as the proportion of female in the total number of adults. Results were shown as mean ± SEM. Student’s *t* test or Mann-Whitney U test was used to compare the pupal time, adult emergence time, wing length, survivorship and sex ratio. *P* > 0.05 indicates no significant difference.

### Impact of *w*Pip on the longevity of HP1 line

**Fig 3:**
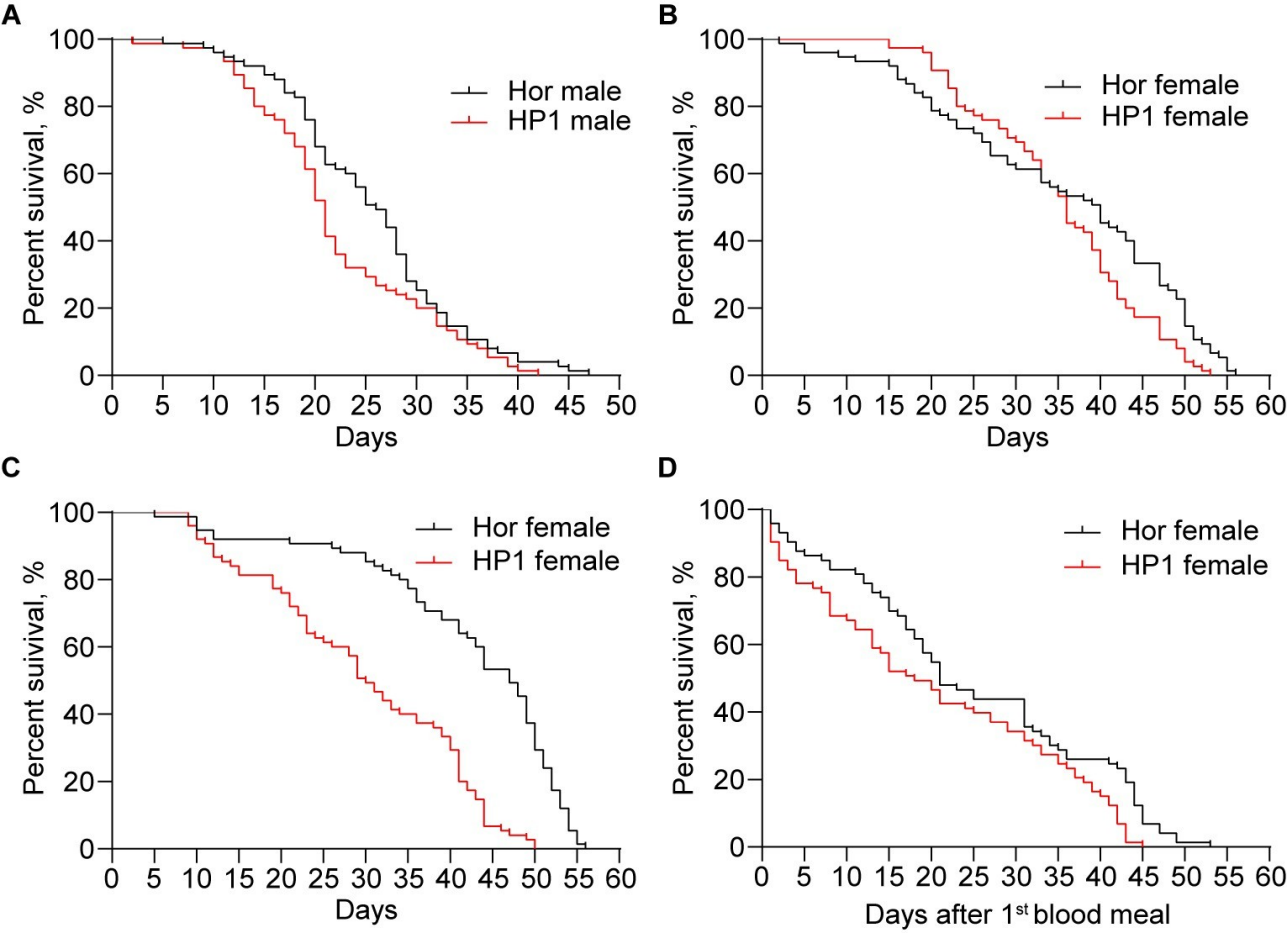
The impacts of *w*Pip on the longevity of HP1 line. (A) The survival curves of unmated males, provided with sugar water only. (B) The survival curves of unmated females, provided with sugar only. (C) The survival curves of mated females, provided with sugar only. (D) The survival curves of mated females, privided with both sugar and blood meal. The dead mosquitoes were removed with an aspirator and recorded daily after the first blood meal. The curves represent the mean percentage of mosquitoes surviving from three biological replicates each day. Log-rank test was used to analyze significant differences in the longevity of adult mosquitoes between HP1 line and Hor line. *P* < 0.05 for A to D.

In order to determine whether *w*Pip impacts the lifespan of HP1 mosquitoes, we compared the longevity of HP1 mosquitoes and Hor mosquitoes under different rearing conditions. When unmated mosquitoes were provided with sugar solution only, both males (Fig 3A, Log-rank test, χ^2^ = 4.28, df = 1, *P* = 0.0385) and females (Fig 3B, Log-rank test, χ^2^ = 4.88, df = 1, *P* = 0.0272) of HP1 line showed a significantly decreased lifespan as compared to the counterparts of Hor line. The median lifespan was 21 days for HP1 males and 26 days for Hor males, while it was 36 days for HP1 females and 40 days for Hor females. Interestingly, HP1 females appeared to survive better during the first 30 days; however, this trend reversed subsequently, with Hor females demonstrating superior survival thereafter (Fig 3B). We then measured the lifespan of mated females by providing with sugar solution only and observed that the HP1 females had the median lifespan 30 days, significantly lower than Hor females, with the median lifespan 47 days (Fig 3C, Log-rank test, χ2 = 50.87, df = 1, P < 0.0001). We further assessed the lifespan of the mated females by providing with both bloodmeal and sugar solution. Again, the HP1 females had the median lifespan 18 days after the first blood meal, significantly lower than Hor females, with the median lifespan 21 days (Fig 3D, Log-rank test, χ2 = 6.77, df = 1, *P* = 0.0093). Taken together, *w*Pip decreases the lifespan of *An*. *stephensi* in all the three rearing conditions.

### Impact of *w*Pip on the reproduction of HP1 line

**Fig 4:**
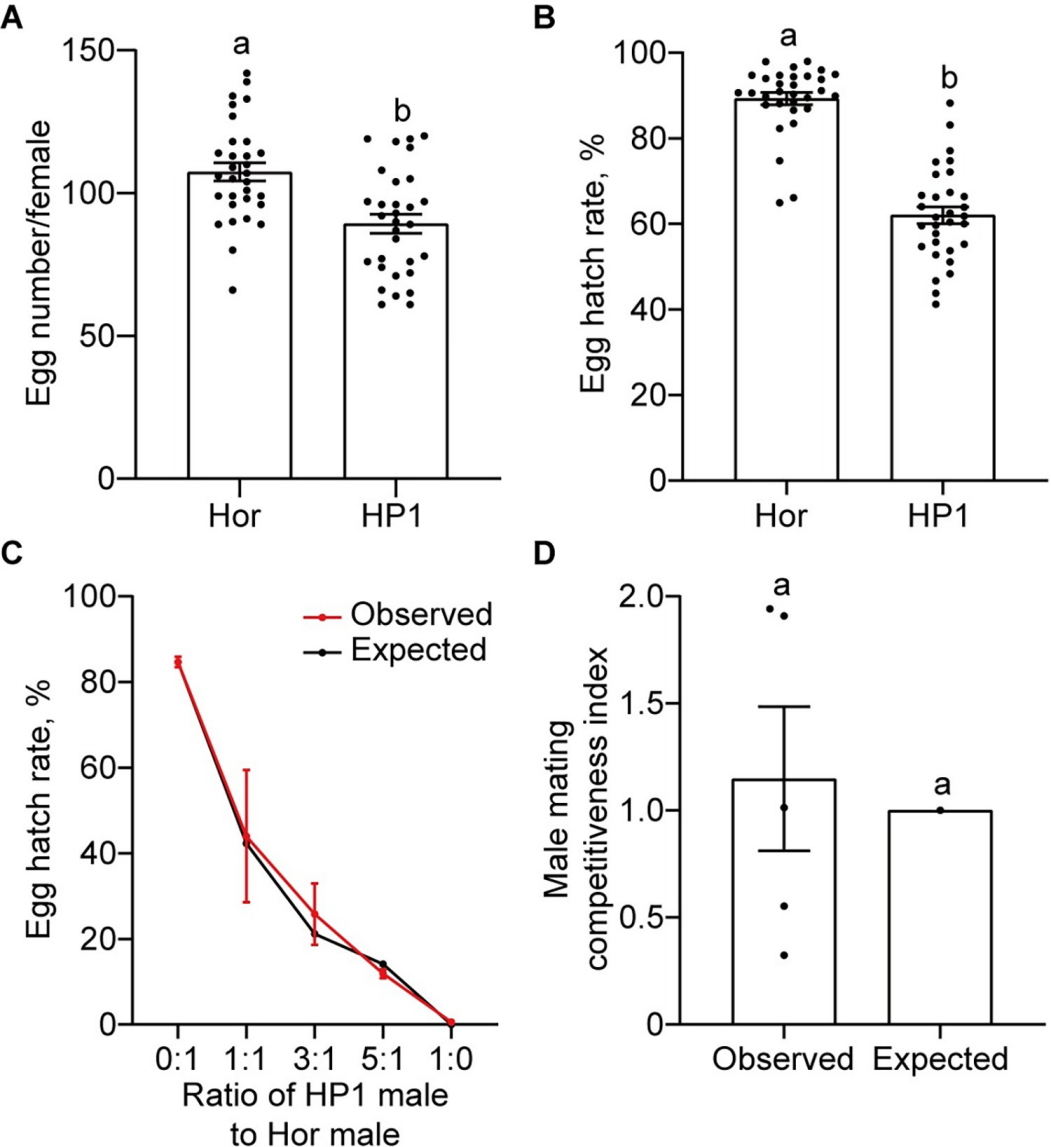
The impact of *w*Pip on the reproduction of HP1 line. (A) The fecundity of HP1 line and Hor line. (B) The eggs hatch rates of HP1 line and Hor line. (C) Egg hatch rates in laboratory cage populations with different Hor female: HP1 male: Hor male ratios. The red line illustrates the observed egg hatching rates, while the black line illustrates the expected egg hatching rates, assuming equal mating competitiveness of HP1 and Hor males and random mating. (D) HP1 male mating competitiveness index. Error bars represent the standard errors based on the biological replicates (*n* = 31 for A and B, *n* = 3 to 5 for C, and *n* = 5 for D). Different lowercase letters above each column indicate significant differences in values, *P* < 0.05, Student’s *t* test for A and B, One sample *t* test for C and D.

To investigate whether *w*Pip influences the reproduction of HP1 line, we compared the fecundity (the number of eggs laid by a female) and fertility (egg hatch rate) of females between HP1 and Hor lines. The results show HP1 females produced a significantly lower number of eggs (89.2 ± 3.3) than Hor females (107.4 ± 3.1) (Fig 4A, Student’s *t* test, *P* < 0.05), and the hatch rates of eggs produced by HP1 females (62.0 ± 1.9%) also were significantly lower than Hor females (89.2 ± 1.4%) (Fig 4B, Student’s *t* test, *P* < 0.05), likely due to partial self-incompatibility.

We then examined the impact of *w*Pip on the competitiveness of HP1 males to mate with Hor females relative to Hor males. Five (1:1) or three cages (0:1, 3:1, 5:1, and 1:0) containing Hor females and different ratios of HP1 males to Hor males were set up. We found that the egg hatch rate was decreased as the ratio of HP1 males increased in the cages (Fig 4C). Consistent with the CI induction, near no egg hatched (0.59 ± 0.17%) at the ratio 1:0, where only HP1 males mated with Hor females. There were no significant differences between the observed egg hatch rates and the predicted values assuming equal competitiveness of HP1 and Hor males and random mating (Fig 4C, One sample *t* test, *P* > 0.05). The male mating competitiveness index at the 1:1 ratio of HP1 males to Hor males was 1.148 ± 0.34, not significantly different from 1 (Fig 4D, one sample *t* test, *P* = 0.4393).

### Tolerance of HP1 mosquito to high temperatures

**Fig 5:**
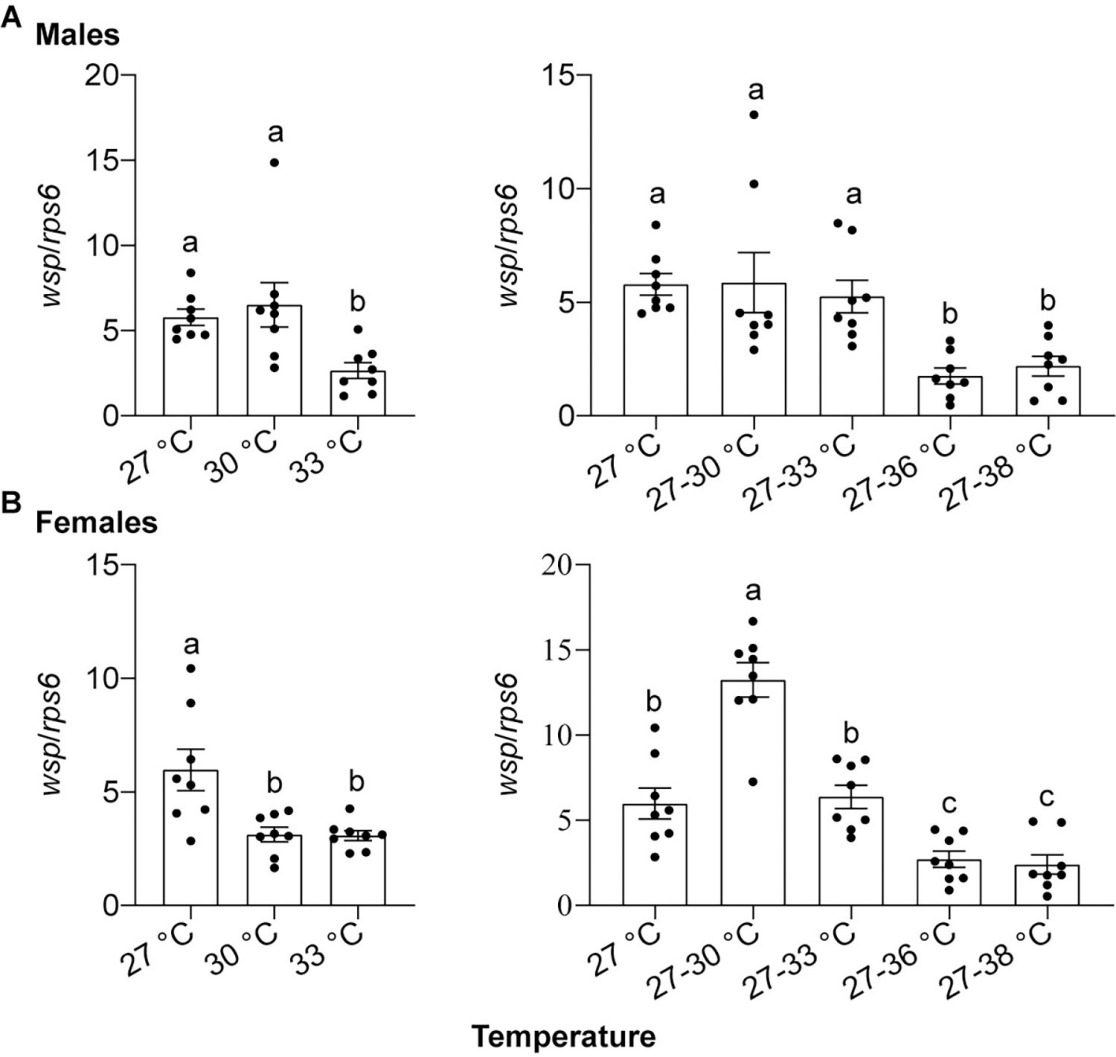
The tolerance of HP1 line to high temperatures. *Wolbachia* densities of male (A) and female (B) in HP1 adults after larvae exposure to either constant temperatures or different diurnal cyclic temperatures. The constant temperature of 27 ± 1°C is used as the control group. *wsp* and *rps6* genes are used as target gene and host reference gene, respectively. Error bars represent the standard errors based on eight biological replicates. Different lowercase letters above each column indicate significant differences in values, *P* < 0.05 (ANOVA analysis, followed by Tukey post-hoc tests).

To investigate the tolerance of *w*Pip-*Anopheles* symbiosis to high temperature, HP1 and Hor larvae were exposed to constant temperatures of 30°C, 33°C, and 27°C (control) and diurnal cyclic temperatures of 27–30°C, 27–33°C, 27–36°C, or 27–38°C. The results showed *Wolbachia* infection frequency maintained 100% in HP1 females and males under all of constant and cyclic temperatures. The *Wolbachia* density in HP1 males exposed to the constant temperatures of 30°C as larvae did not differ significantly from the control 27°C; however, the *Wolbachia* densities in HP1 males exposed to 33°C as larvae were significantly lower than in those exposed to 30°C and 27°C as larvae (Fig 5A, ANOVA analysis, F_2, 21_ = 5.875, *P* < 0.05). At the diurnal cyclic temperatures, there was no significant difference in *Wolbachia* densities among HP1 males exposed to 27°C, 27–30°C, 27–33°C as larvae; however, while HP1 males exposed to temperatures of 27–36°C and 27–38°C as larvae exhibited similar *Wolbachia* densities, these densities were significantly lower compared to other groups (Fig 5A, ANOVA analysis, F_4, 35_ = 7.320, *P* < 0.05). Furthermore, HP1 females exposed to 30°C and 33°C as larvae exhibited significantly lower *Wolbachia* densities than that exposed to 27°C as larvae (Fig 5B, ANOVA analysis, F_2, 21_ = 8.515, *P* < 0.05). Under the diurnal cyclic temperatures, HP1 females exposed to 27–36°C and 27–38°C as larvae also had significantly lower *Wolbachia* densities than those exposed to 27–30°C, 27–33°C and 27°C as larvae; by contrast, HP1 female exposed to 27–30°C as larvae had the highest *Wolbachia* densities among all the tested groups (Fig 5B, ANOVA analysis, F_4, 35_ = 33.08, *P* < 0.05). No difference in *Wolbachia* densities was observed between 30°C and 33°C, between 27–36°C and 27–38°C, and between 27°C and 27–33°C (Fig 5B). These results suggest that *w*Pip densities in *An*. *stephensi* may decrease as environmental temperatures reach sufficiently high levels. However, this transinfection remains robust to a certain extent, maintaining a 100% infection frequency even under elevated temperatures.

In addition to *Wolbachia* densities, we also examined whether high larvae rearing temperature stress might differ in their impacts on the development of HP1 and Hor lines. We found that both HP1 and Hor lines exhibited a decrease in pupae and adult emergence rates with the increase of larvae rearing temperature to 33°C, 36°C, 38°C, 27–36°C, and 27–38°C compared to 27°C and other treatments (S2 Fig). However, under the same rearing temperatures, even at high larvae rearing temperatures, there were no differences in the pupation rates and adult emergence rates between HP1 and Hor lines (S2 Fig).

## Discussion

In this study, we have succeeded in transferring *Wolbachia w*Pip into *An*. *stephensi* and established a stable maternally inherited artificial infection, which is the second *Wolbachia* transinfection in *Anopheles* mosquitoes. We show that *w*Pip in *An*. *stephensi* induces nearly complete unidirectional CI and distributes in both somatic and germline tissues. There is no impact of *w*Pip on immature survivorship and development time, sex ratio, body size and male mating competitiveness. By contrast, *w*Pip reduces both the life span of transinfected mosquito and female reproduction capacity. While exposing larvae to high temperature reduces the densities of *w*Pip and negatively affects immature development, *Wolbachia* infection frequence has been stably maintained at 100% in the transinfected line and there is no difference in immature tolerance to high temperature between transinfected and wild-type lines. These results support the robustness of the *w*Pip transinfection in *Anopheles* with only moderate fitness cost detected and highlight its potential to be used for malaria control in endemic countries.

The generation of the 2nd transinfection in *An*. *stephensi* allows us to analyze the phenotypes induced by these two supergroup B strains, *w*Pip and *w*AlbB. However, direct comparison should be avoided as they were studied in separate experiments. Both strains induce near complete CI, with egg hatch rates in the CI crosses 0.8% and 1.2% for *w*Pip and *w*AlbB [20], respectively. The self-crosses of both transinfected lines show reduced egg hatch compared to the other two compatible crosses, which might be caused by partial self-incompatibility or a transinfection-associated cost on female reproduction rather than inbreeding effects. This is evidenced by the persistence of reduced hatching after outcrossing with wild types for six generations and by normal egg hatch rates observed in crosses between transinfected females and wild-type males. Similar to *w*AlbB, *w*Pip has the highest density in fat bodies and lowest in midguts among the tested tissues, different with their native infection, typically highly enriched in ovaries related to somatic tissues [37]. Both *w*Pip and *w*AlbB have no impact on the life history traits and sex ratio of the transinfected lines, with either no or very minor impact on male mating competitiveness [35]. While *w*AlbB increased the longevity of *An*. *stephensi* when mosquitoes were provided with sugar alone [35], *w*Pip consistently reduced mosquito life span when providing with either sugar alone or blood meal. In general, although the two transinfected lines were generated from different host backgrounds in either the LIS or Hor strain [20,32], they exhibit more similarities than differences, indicating the presence of conserved interactions between *Wolbachia* supergroup B and *Anopheles* hosts. Given that *w*AlbB induces resistance to both *P*. *berghei* [25] and *P*. *falciparum* [20], while *w*Pip does not inhibit dengue viruses in transinfected *Aedes aegypti* [38], it would be informative to investigate the impact of *w*Pip on vector competence for malaria parasites. Furthermore, a previous report shows no relationship between *Wolbachia* density and the blocking of parasites [39], calling for further investigation into the *Anopheles-Wolbachia-Plasmodium* interactions.

Although not evaluated in *w*AlbB-infected *An*. *stephensi*, we investigated the tolerance of the *w*Pip-transinfected HP1 line to heat stress during the immature stage, given *Anopheles* larvae can still be found alive when the water temperature at the breeding habitat reaches 36°C [40] in malaria endemic areas, and the temperature sensitivity of *Wolbachia*-mosquito associations observed in the transinfected *Aedes* mosquitoes [41–43]. Importantly, none of the high larvae rearing temperature treatments resulted in loss of *Wolbachia* in transinfected line. For both males and females, we observed a consistent reduction in *w*Pip densities when larvae were exposed to sufficiently high temperatures, including a constant temperature of 33°C, as well as to diurnal cyclic temperatures of 27–36°C and 27–38°C. However, upon exposure to 30°C, *w*Pip densities decreased in females but remained unchanged in males. Additionally, cyclic temperatures of 27–30°C did not affect *w*Pip densities in males, yet increased them in females. These findings suggest a gender-specific difference in regulation of *w*Pip densities by temperature.

Heat stress is reported to have negative, strain-specific effects on CI and *Wolbachia* load [41–43]. Increased temperatures are associated with a reduced expression of CI and lowered *Wolbachia* density, and this trend appears to vary by strain in the transinfected *Ae*. *aegypti* (e.g., more marked in *w*Mel than in *w*AlbB) [43]. As a nutrition-consuming parasite, *Wolbachia* increases the mosquito’s energy requirements, and there is likely a trade-off between this increased energy requirement and mosquito fitness at increasing temperatures [44]. Although field trials have demonstrated that the *w*Mel strain can achieve *Ae*. *aegypti* population replacement and dengue control in some regions, such as Australia and Indonesia [21,30], this same *Wolbachia* strain struggles to spread in other areas, such as Vietnam and Brazil [45,46], pointing to temperature and other environmental conditions as the key drivers for the low efficacy in certain settings. Field populations experience variable temperatures and periods of rainfall, as well as limited food resources available to larvae, likely leading some *Wolbachia* strains to parasitize mosquitoes by depleting their energetic resources and reducing their fitness, as observed in *w*MelPop- and *w*AlbB- transinfected *Ae*. *aegypti* [47]. In addition, both *w*AlbB and *w*Mel are reported to reduce the thermal tolerance of *Ae*. *aegypti* [43,48]. However, we have not observed increased sensitivity to high temperatures in the immature development and survivorship of the HP1 line relative to its wild counterpart. *An*. *stephensi* is reported to survive extremely high temperatures during the dry season, when malaria transmission usually reaches a seasonal low. Thus, future studies are needed to assess how these stressful field conditions will affect *Wolbachia* densities and CI expression, and compare how known environmental factors affect *Wolbachia-Anopheles-Plasmodium* interactions across different *Wolbachia* strains.

The expansion of *An*. *stephensi* has triggered a recent WHO initiative aimed at determining whether this vector can be eradicated from regions it has already established. The successful application of the Incompatible Insect Technique (IIT) for eliminating the invasive mosquito species *Aedes albopictus* [26], combined with the lack of negative impact of *w*Pip on the mating competitiveness of HP1 males observed in this study, supports the potential of releasing incompatible males to suppress and eliminate *An*. *stephensi* populations. However, it is more challenging to achieve mass production and sex separation in *Anopheles* mosquitoes compared to *Aedes* mosquitoes. We noted a *w*Pip-associated fitness cost affecting female reproduction and adult lifespan. Such reductions in female fecundity and fertility may increase the cost of mass-rearing the transinfected line. In strategies involving population replacement, these fitness costs could increase the threshold frequency of *Wolbachia* required for successful replacement. However, once replacement is achieved, these costs could potentially facilitate disease control by both lowering mosquito density and reducing the probability of mosquitoes surviving through the extrinsic incubation period. The variability of these fitness costs with environmental conditions and mosquito genetic backgrounds, and their potential impacts on the spread of *w*Pip in field populations, remain to be explored.

In conclusion, we have successfully established *w*Pip transinfection in *An*. *stephensi*. The ability of *w*Pip to achieve perfect maternal transmission, induce nearly complete CI, and exhibit no measurable fitness cost on the immature development and male mating competitiveness in the HP1 line while maintaining reduced infection densities at temperatures up to 38°C supports further evaluation of the HP1 line for malaria control. Future studies should investigate the *w*Pip-mediated *Plasmodium* blocking effect, its potential to induce population replacement and suppression, and the long-term stability of *Wolbachia-Anopheles* associations under real-world field conditions.

## Supporting information

S1 Table PCR-screening of *w*Pip infection in the G1 offsprings developed from the nine *w*Pip-positive G0 isofemale lines. (DOCX)

S2 Table *w*Pip positive rates in the PCR-screening of HP1 line from G1 to G10. (DOCX)

S3 Table Cytoplasmic incompatibility (CI) between the HP1 line and Hor line. (DOCX)

S1 Fig Representative PCR results for validation of the perfect *w*Pip maternal transmission in HP1 line at different generations. (TIF)

S2 Fig The impact of high temperatures on the immature development of HP1 line and Hor line. (TIF)

S1 Data Excel spreadsheet containing, in separate sheets, the underlying numerical data and statistical analysis for Table 2 and Figs 1, 2A, 2D, 2E, 3A–3D, 4A–4D, 5A, 5B, S2A and S2B of the study. (XLSX)
