# Supplementary material for: Stable introduction of Wolbachia wPip into invasive Anopheles stephensi for potential malaria control
Source: PLoS Negl Trop Dis. 2024 Sep 26;18(9):e0012523. doi: 10.1371/journal.pntd.0012523 (PMC11460690; doi:10.1371/journal.pntd.0012523)
Supplement: S1 Table — (DOCX) [file pntd.0012523.s001.docx]

**Table S1. PCR-screening of *w*Pip infection in the G1 offsprings developed from the nine *w*Pip-positive G0 isofemale lines.**

| *Wolbachia*  donor | Recipient  mosquito | G0 positive  isofemale line | %, *Wolbachia* frequency | |
| --- | --- | --- | --- | --- |
|  |  |  | G1 males | G1 females |
| *w*Pip | Hor | 1 | 31.6 (6/19) | 30 (3/10) |
|  |  | 2 | 0 (0/15) | 0 (0/8) |
|  |  | 3 | 0 (0/20) | 0 (0/10) |
|  |  | 4^#^ | - | - |
|  |  | 5^#^ | - | - |
|  |  | 6^#^ | - | - |
|  |  | 7^#^ | - | - |
|  |  | 8^#^ | - | - |
|  |  | 9^#^ | - | - |

^#^ means G0-positive isofemales did not produce any progeny.
