# Supplementary material for: Stable introduction of Wolbachia wPip into invasive Anopheles stephensi for potential malaria control
Source: PLoS Negl Trop Dis. 2024 Sep 26;18(9):e0012523. doi: 10.1371/journal.pntd.0012523 (PMC11460690; doi:10.1371/journal.pntd.0012523)
Supplement: S2 Table — (DOCX) [file pntd.0012523.s002.docx]

**Table S2. *w*Pip positive rates in the PCR-screening of HP1 line from G1 to G10.**

| **Generation** | 1 | 2 | 3 | 4 | 5 | 6 | 7 | 8 | 9 | 10 |
| --- | --- | --- | --- | --- | --- | --- | --- | --- | --- | --- |
| **%,**  **Females** | 30.0  (3/10) | 60.0  (3/5) | 62.5  (5/8) | 90.0  (9/10) | 75.0  (12/16) | 50.0  (8/16) | 75.0  (12/16) | 95.7  (22/23) | 100.0  (18/18) | 100.0  (16/16) |
| **%,**  **Males** | 31.6  (6/19) | 71.4  (5/7) | 50.0  (4/8) | 50.0  (5/10) | 81.3  (13/16) | 62.5  (10/16) | 81.3  (13/16) | 100.0  (23/23) | 100.0  (18/18) | 100.0  (16/16) |
