# Supplementary material for: Stable introduction of Wolbachia wPip into invasive Anopheles stephensi for potential malaria control
Source: PLoS Negl Trop Dis. 2024 Sep 26;18(9):e0012523. doi: 10.1371/journal.pntd.0012523 (PMC11460690; doi:10.1371/journal.pntd.0012523)
Supplement: S3 Table — (DOCX) [file pntd.0012523.s003.docx]

**Table S3. Cytoplasmic incompatibility (CI) between the HP1 line and Hor line**

| Potential  CI Type | Cross | Infection type | | %, egg hatch rate  （mean ± SEM） | Eggs |
| --- | --- | --- | --- | --- | --- |
|  | Female × Male | Female | Male |  |  |
| Unidirectional | HP1 × Hor | *w*Pip | - | 75.5 ± 3.1 | 1957 |
|  | Hor ×HP1 | - | *w*Pip | 0.8 ± 0.1 | 1992 |
| Compatible | HP1 ×HP1 | *w*Pip | *w*Pip | 62.9 ± 5.2 | 2381 |
|  | Hor × Hor | - | - | 79.6 ± 1.7 | 2240 |
