# Supplementary figures and images for: Stable introduction of Wolbachia wPip into invasive Anopheles stephensi for potential malaria control
Source: PLoS Negl Trop Dis. 2024 Sep 26;18(9):e0012523. doi: 10.1371/journal.pntd.0012523 (PMC11460690; doi:10.1371/journal.pntd.0012523)

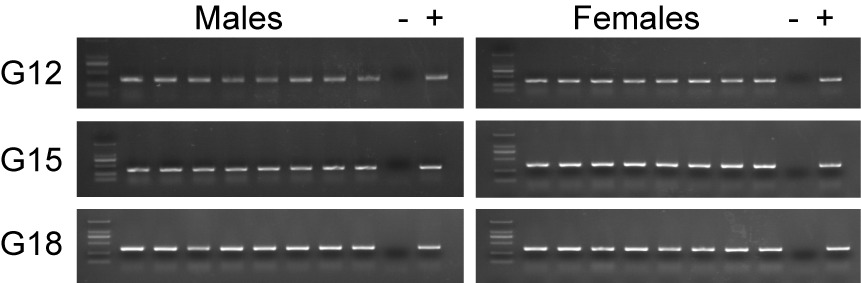

Supplement: S1 Fig — (TIF) [file pntd.0012523.s004.tif]

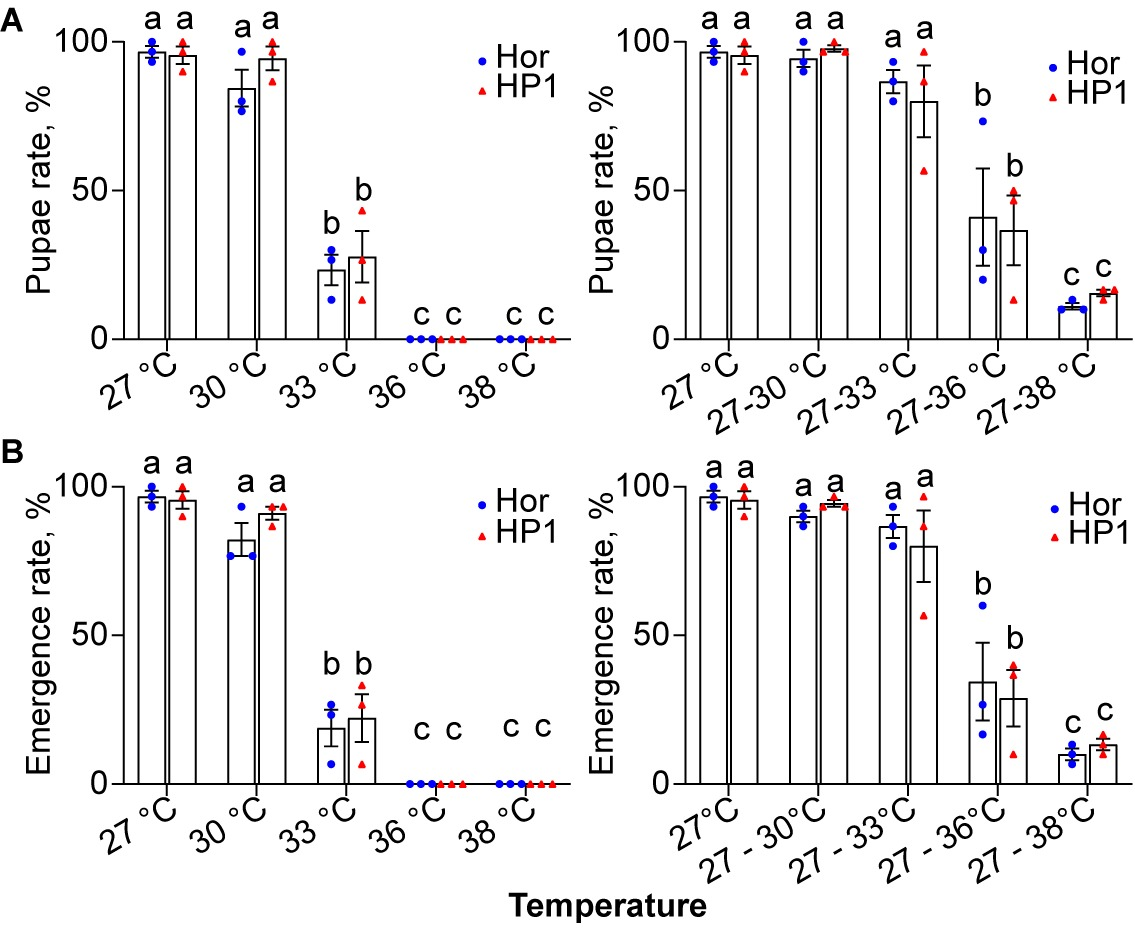

Supplement: S2 Fig — (TIF) [file pntd.0012523.s005.tif]
